# Impact of sample handling protocols on soft tissue attenuation coefficient and morphology

**DOI:** 10.1117/1.JBO.30.10.106004

**Published:** 2025-10-14

**Authors:** Freja Hoeier, Gavrielle R. Untracht, Amanda Oester Andersen, Karina Straede, Andreas Kjaer, Peter E. Andersen

**Affiliations:** a Technical University of Denmark, Health Tech, Biophotonic Imaging Group, Kongens Lyngby, Denmark; b Copenhagen University Hospital, University of Copenhagen, Rigshospitalet & Department of Biomedical Sciences, Department of Clinical Physiology, Nuclear Medicine & PET and Cluster for Molecular Imaging, Copenhagen, Denmark; c Copenhagen University Hospital, Department of Otorhinolaryngology, Head & Neck Surgery and Audiology, Rigshospitalet, Denmark

**Keywords:** tissue optics, sample handling, optical coherence tomography, frozen, fresh, tissue attenuation coefficient

## Abstract

**Significance:**

The use of tissue attenuation coefficients as biomarkers for disease detection is rising. However, especially for *ex vivo* studies, sample handling methods can notably impact tissue optical attenuation properties, and these effects have yet to be studied in detail.

**Aim:**

We aim to compare and evaluate common methods for sample handling and assess their impact on the optical attenuation and structural properties of *ex vivo* colon tissue.

**Approach:**

Six different handling methods were tested: Direct freezing at −80°C, slow freezing in a cryobox with and without cryopreservation media, snap freezing in isopentane, formalin fixation, and fresh tissue stored directly in phosphate-buffered saline. All samples were imaged using optical coherence tomography; images were assessed qualitatively for morphological changes and quantitatively by extracting the tissue attenuation coefficient using the Lambert–Beer law. All handling methods were compared with representative histology (hematoxylin and eosin staining and periodic acid–Schiff staining).

**Results:**

All sample handling methods showed a significant difference in tissue attenuation and morphology relative to the fresh tissue (p≪0.0001), with frozen samples generally showing a lower attenuation coefficient, e.g., directly frozen ($2.0\pm 1.0\;{\mathrm{mm}}^{-1}$) compared with formalin-fixed ($2.5\pm 1.3\;{\mathrm{mm}}^{-1}$) and fresh tissue ($2.5\pm 1.0\;{\mathrm{mm}}^{-1}$). Formalin-fixed and snap frozen samples had the smallest effect size (δ=0.002 and −0.09, respectively). Macroscopic structural changes were also observed, including alterations to the epithelial layer and indications of goblet cell degradation for all methods but formalin fixation.

**Conclusions:**

Understanding the impact of sample handling methods is critical to the accurate interpretation of morphology-based analysis. In the case of fresh tissue being unavailable, formalin-fixed and snap frozen tissue samples yield the best alternative with negligible effect sizes for colon tissue.

## 1 Introduction

Alterations in tissue, such as the onset of disease, have been observed to affect the absorption and scattering properties—which, in turn, change the attenuation of light or sound waves propagating in the tissue.^1^ In computed tomography (CT) and ultrasound, tissue attenuation is used to differentiate among tissue types, providing detailed insights into tissue structure and function.^2^^,^^3^ In CT, the tissue attenuation is measured by the loss of intensity of the X-ray beam passing through the sample, mainly due to water absorption.^2^^,^^4^ However, contrast in CT is limited by the small difference in absorption coefficient among soft tissues, making it better suited for higher density tissues such as bone.^2^ Ultrasound is sensitive to the decrease in the amplitude of the acoustic wave propagating through tissue, which is affected by not only absorption but also scattering, reflection, refraction, and diffraction.^3^ The change in wave amplitude enables better distinction among tissues with similar density. However, the resolution of ultrasound does not currently allow for the identification of microscopic changes in tissue, which is important in satellite lesion detection and *in vivo* margin assessment.^3^

To address the limited resolution and sensitivity of conventional medical imaging techniques in tissue attenuation measurements, optical imaging techniques have been investigated. Optical coherence tomography (OCT) stands out as a promising approach.^5^^–^^7^ OCT is based on optical scattering contrast and provides a label-free volumetric representation of tissue microstructure.^5^^,^^8^ OCT can acquire real-time images over a large field of view of several mm with an imaging resolution in the range of 2 to 10  μm and an imaging depth up to 1 to 2 mm.^5^^,^^8^

Several recent studies have shown the strong promise of OCT-derived attenuation coefficients to aid in the differentiation of diseased tissue. Turani et al.^9^ showed that OCT can provide information indicative of changes in tissue microstructure and aid in the identification of melanoma when used in conjunction with computer learning algorithms. Foo et al.^10^ and McLaughlin et al.^11^ highlighted the role of the attenuation coefficient in breast cancer detection, whereas van der Meer et al.^12^ illustrated the potential of using the attenuation coefficients for detection of atherosclerotic plaque. In these studies, tissue-attenuation-coefficient-based methods were developed for diagnostic purposes. As many of these studies used soft *ex vivo* tissue samples,^10^^–^^13^ it is important to understand the effects of sample preparations on tissue attenuation. This highlights the need for a systematic investigation of different possible sample handling methods.

The most optimal protocol would be to image fresh tissue immediately after surgical resection to minimize degradation time. However, when including transportation of the samples from the extraction site to the imaging site, a further time constraint is added to the imaging process. Studies have shown that keeping fresh tissue at 5°C prolongs the usable lifetime of the sample. Despite this, samples still have a limited lifetime during which they remain useful.^14^ In addition, we must be mindful of the ethical use of animals for experiments by minimizing the number of animals used and by acquiring multiple samples from the same animal whenever possible. As it is challenging to perform multiple experiments simultaneously, this underscores the need for long-term storage. Therefore, many studies utilize samples that have undergone some additional processing to prolong their lifetime. For example, freezing is a common method for longer term storage, although it has long been known that the freezing process can have a detrimental impact on tissue structure.^15^^,^^16^ Indeed, specialized tissue freezing protocols have been developed to minimize damage to morphological structures caused by freezing,^15^^,^^17^^–^^19^ but they do not necessarily protect tissue from damage induced by ice crystallization.

Some preliminary studies have therefore used OCT imaging to investigate changes in soft tissue optical properties due to freezing. Li et al.^20^ and Kropatsch et al.^21^ compared fresh samples with frozen samples (slowly frozen;^20^ slowly frozen, and snap frozen^21^) to investigate changes in tissue attenuation. These studies both showed a difference between fresh and frozen samples. However, these studies only compared 1 to 2 freezing methods with fresh samples; they did not consider other common tissue preservation techniques and did not compare the results with the gold standard (i.e., histology).

In this study, we perform a systematic comparison of the impact of freezing methods (snap frozen in isopentane, directly frozen at −80°C, slowly frozen in a cryobox with and without cryopreservation media) and formalin fixation based on tissue attenuation coefficient and morphological changes of colon tissue.^22^ To further understand possible differences, all results will be compared with the gold standard, i.e., histological staining. Our study focuses on colon tissue as our soft tissue model. Colon tissue is a soft, heterogenous tissue type that contains cells with high water concentration, e.g., goblet cells. We expect to see a larger impact on the colon tissue from freezing methods compared with relatively homogeneous tissues with fewer water-containing cells.^15^^,^^21^ This complexity makes colon tissue a relevant model for testing and optimizing sample handling protocols.

## 2 Method

### 2.1 Sample Handling

All animal procedures were in accordance with protocols approved by the Danish Animal Experiment Inspectorate (License No. 2021-15-0201-01041). In this study, distal colon tissue samples were collected from 17 healthy mice (Rj:NMRI-${\mathrm{Foxn}1}^{\mathrm{nu}/\mathrm{nu}}$) aged 8 to 11 weeks. This age difference is due to the mice being euthanized as part of other studies.

The dissected colon samples were cut into lengths of 1 to 1.5 cm, and then each section was cut open longitudinally to reveal the epithelium. For each of the preparation methods, three samples from different mice were collected to account for biological variation among mice. For the fresh method, five distal colon tissue samples were used instead of three.

Each tissue sample was prepared in one of the six ways to represent different common preparation methods^14^^,^^17^^–^^19^^,^^21^^–^^24^:

• **Direct:** Directly put in an Eppendorf tube and into −80°C freezer immediately after dissection.
• **Slow:** Inserted in a cryobox and thereafter put in a −80°C freezer (controlled decrease of temperature of 1°C/min).
• **DMSO:** Submerged in cryopreservation media [Dulbecco’s Modified Eagle Medium (DMEM) + 10% fetal bovine serum +10% dimethyl sulfoxide (DMSO)], inserted in a cryobox and put in a −80°C freezer (controlled decrease of temperature of 1°C/min).
• **Snap:** Snap frozen by being directly submerged in isopentane on dry ice; thereafter stored in at −80°C until imaging.
• **Formalin:** Submerged in 4% formaldehyde for 24 h in a tissue holder (to keep the colon unfolded), cleaned with 70% ethanol, and stored in phosphate-buffered saline (PBS) at 5°C until imaging.
• **Fresh:** Submerged in PBS. Kept at 5°C until imaging, which happened within 2 h of extraction.

All frozen samples were thawed for 5 min at room temperature in PBS. All samples were imaged submerged in PBS to keep the samples hydrated and to reduce the impact of surface reflections on the analysis. Due to the colon floating on the PBS surface, pins were used to keep the sample in place during the imaging. The imaging session took ∼30  min for each sample.

### 2.2 OCT Imaging

OCT images were acquired using a spectral domain OCT system (TELESTO II, Thorlabs Inc., Newton, New Jersey, United States) with a center wavelength of 1310 nm and a magnification of 5× (LSM03, Thorlabs Inc., Newton, New Jersey, United States). The system had an axial resolution of 5.5  μm (in air) and a lateral resolution of 13  μm (in air). A line-scan rate of 28 kHz was used. Images were acquired over a volume of $3\times 2\times 3\;{\mathrm{mm}}^{3}$ ($852\times 568\times 852\;{\mathrm{pixel}}^{3}$) with a pixel size of 3.52  μm (in air) in both the axial and lateral directions. On each sample, 3 to 5 locations were imaged ∼0.5  mm apart to obtain an equal distribution of samples along the distal colon. The precise number of imaging locations depended on the size of the colon sample extracted from the mouse.

### 2.3 Histology Preparation

Histology samples were prepared at varied times before fixation to simulate the imaging procedure. The “formalin” sample was fixed using 4% formaldehyde directly after dissection. The “fresh” sample was stored in PBS for 2 h, whereas the frozen samples were thawed and stored in PBS for 30 min before all samples were fixed using 4% formaldehyde. After 24 h, all samples were cleaned with 70% ethanol and embedded in paraffin. Then, the samples were cut and stained with either hematoxylin and eosin (H&E) or periodic acid–Schiff (PAS) according to standard protocol. PAS was used as additional staining to highlight the goblet cells in the colon tissue.

Images of the H&E and PAS-stained histology slides were taken using Zeiss Axioscan Z1 with a Plan-Apochromat 10×/0.45 M27 objective and a Hitachi HV-F202SCL camera.

### 2.4 Image Analysis

**Fig. 1:**
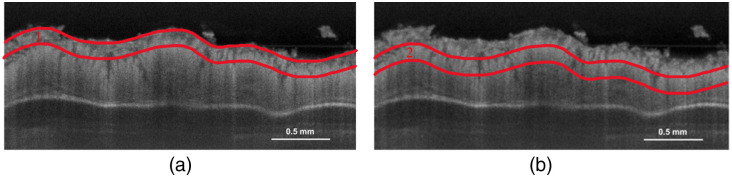
Segmentation of the colon sample into two regions; in each of the two regions, an attenuation coefficient was extracted. Both images are incoherent averaged OCT B-scans comprising 11 scans over 38  μm. The scalebar is 0.5 mm. (a) The first region—defined from the surface of the colon tissue and ∼150  μm into the tissue. (b) The second region—defined 150  μm into the tissue and until 300  μm, covering the middle and end part of the crypts.

Data obtained from the OCT system were processed in MATLAB 2022b (MathWorks, Inc., Natick, Massachusetts, United States). The OCT images were corrected to account for system characteristics, including sensitivity roll-off and confocal point spread function.^25^ As the samples were submerged in PBS during imaging, the focal plane placement and depth axis were corrected assuming a refractive index of 1.33. A peak algorithm (findpeaks) was used to identify the sample surface. A mean filter with a kernel size of 11 pixels (38  μm) was applied in the lateral direction to average out speckle variation in individual A-scans and improve the fitting of the attenuation coefficient. The kernel size was chosen based on a balance between reducing the influence of speckle on the fit precision and preserving spatial information. The fitting of the attenuation coefficient was performed for all A-scans on each of 568 B-scans from each volumetric OCT dataset. In total, ∼43,700 attenuation coefficients were obtained for each 3D volume, leading to ∼600,000 to 1,000,000 attenuation coefficients per handling method. The attenuation coefficient was found by fitting the Lambert–Beer law ($I=I_{0}\exp(-2\mu_{\alpha }d)$ on the logarithmic OCT data in two defined regions, as illustrated in Fig. 1.

The crypts were divided into two areas with a depth range of 50 pixels (150  μm) in each region. The regions were chosen based on the initial analysis of observed morphological differences in the two regions, as displayed in Fig. 1. Region 1 started at the beginning of the crypts (sample surface) and spanned ∼150  μm into the tissue. Region 2 started right after region 1, ∼150  μm inside the colon tissue, and until the end of the crypts, ∼300  μm inside the colon. Both regions lay within a normal crypt size of 150 to 300  μm.^26^

An outlier remover (rmoutliers) was applied using the median setting (three median absolute deviations) to the extracted attenuation coefficients, eliminating any outliers. Subsequently, all negative attenuation coefficients were rejected (see Sec. 3.1). Mean and standard deviations were calculated across both regions. A Mann–Whitney U test (ranksum) was conducted to compare each handling method with the “fresh” sample for both regions. Finally, an effect size measurement was calculated based on Cliff’s delta (meanEffectSize).^27^^,^^28^

For the investigation of morphological changes, the data were preprocessed as described in the extraction of the attenuation coefficient, including a mean filter with a kernel size of 11. However, instead of a peak detection, the surface was detected using a Canny edge detection algorithm, and the tissue was flattened using a circular shift operation to compensate for the curved tissue surface. An incoherent average of 11 *en face* planes was extracted at the depth range 15 to 50  μm from the sample surface. The observed morphological changes in the OCT images were identified by comparison to histology with the guidance of a pathologist.

## 3 Results

### 3.1 Attenuation Coefficient

**Fig. 2:**
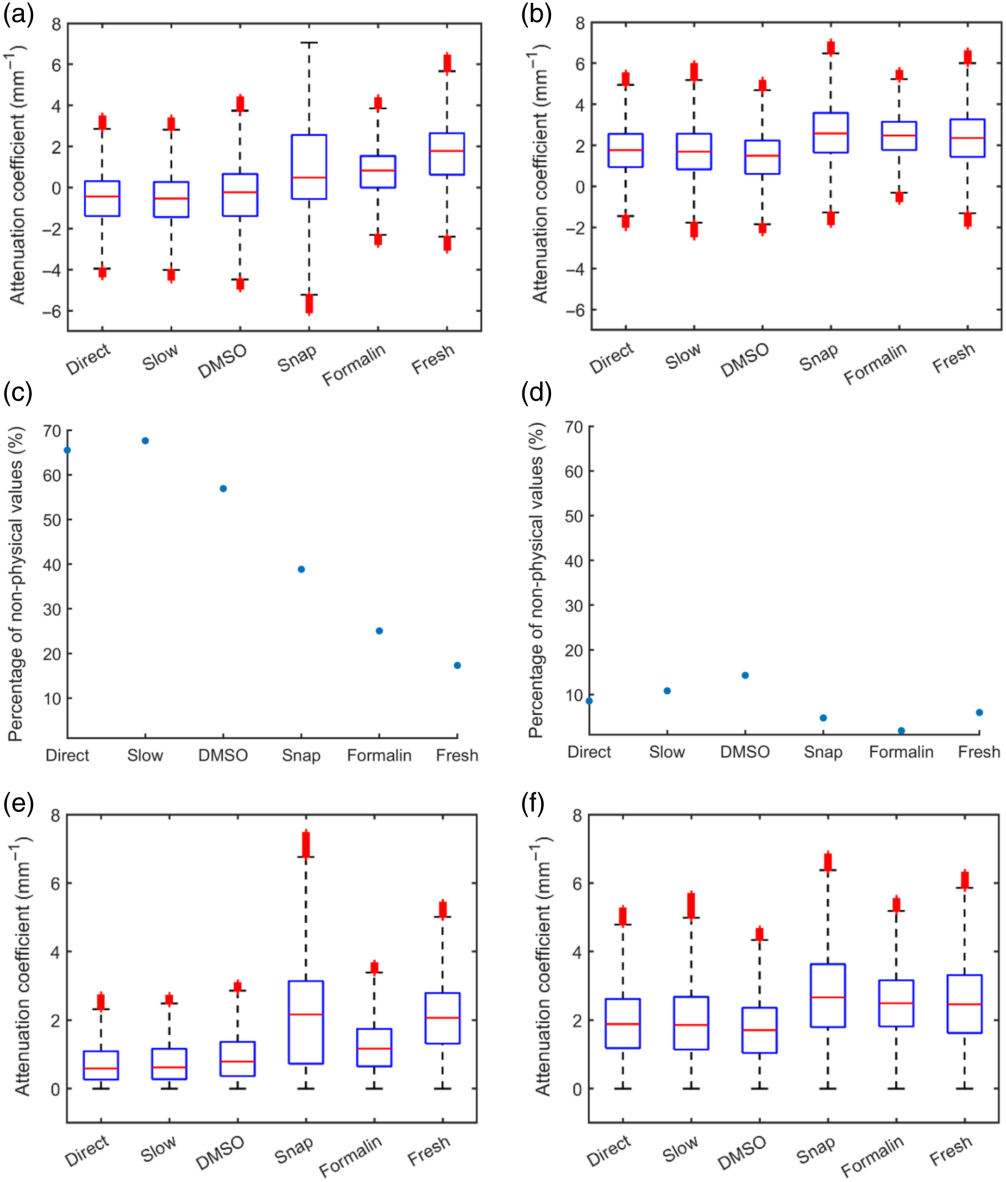
Extracted attenuation coefficients with and without nonphysical values for region 1 (left) and region 2 (right). (a) Attenuation coefficient, including nonphysical values for region 1 (b) Attenuation coefficient, including nonphysical values for region 2. (c) Percentage of nonphysical values observed in the different samples for region 1. (d) Percentage of nonphysical values observed in the different samples for region 2. (e) Attenuation coefficient boxplot without nonphysical values for region 1. (f) Attenuation coefficient boxplot without nonphysical values for region 2. The red line on the boxplots indicates the median, the box is the interquartile range, and the whiskers are the full range. Red crosses beyond the whiskers indicate outliers.

When we applied the Lambert–Beer fitting, we assumed the tissue to be relatively homogeneous in the fitting area; this is a typical assumption in attenuation measurements. However, in cases where this assumption was violated, the fitting does not work correctly, leading to negative values or very high variability in the attenuation [Figs. 2(a) and 2(b)]. The negative attenuation coefficient values, considered nonphysical values, have been displayed here to emphasize the clear difference between regions 1 and 2 in regard to the number of nonphysical values.

A direct observation shows a high percentage of nonphysical values relative to the total number of measured attenuation coefficients in region 1. In particular, higher percentages of nonphysical values were observed for the frozen sample handling methods, with the “direct” method yielding 66% nonphysical values, whereas the “fresh” method yields 17%. As a result, in Fig. 2(e), where all nonphysical values have been removed, there is an exclusion of 66% of the attenuation measurements in the case of the “direct” method. Further elaboration on this can be found in Sec. 4. The same trend regarding the percentage of nonphysical cannot be found in region 2. Here, the “DMSO” method has the highest percentage of nonphysical values (15%), whereas the “fresh” method has the lowest percentage (7%); both numbers are notably lower compared with region 1.

Removal of nonphysical values renders the extracted attenuation coefficient represented by physical values only. From these results, a large variation in attenuation coefficient can be observed with a range of 0 to $8\;{\mathrm{mm}}^{-1}$ depending on the handling method. The boxplots in Fig. 2 illustrate how the frozen handling methods, except “snap”, trend toward a lower attenuation coefficient compared with the “fresh” sample. Region 2 has a higher attenuation coefficient compared with region 1, see also Table 1.

**Table 1:** Average extracted attenuation coefficient reported as mean ± standard deviations for all six methods for both regions. The P-values were obtained from the Mann–Whitney U test and Cliff’s delta effect size with “fresh” samples as the control group for both regions.

		Direct	Slow	DMSO	Snap	Formalin	Fresh
**Region 1**	**Attenuation coefficient (mm^−**1**^)**	0.8±0.6	0.8±0.6	0.9±0.7	2.1±1.4	1.2±0.8	2.1±1.1
	***p*** **-value**	p≪0.0001	p≪0.0001	p≪0.0001	p≪0.0001	p≪0.0001	—
	**Effect size**	0.71	0.69	0.62	0.008	0.47	—
**Region 2**	**Attenuation coefficient (mm^−**1**^)**	2.0±1.0	2.0±1.2	1.7±0.9	2.8±1.4	2.5±1.3	2.5±1.0
	***p*** **-value**	p≪0.0001	p≪0.0001	p≪0.0001	p≪0.0001	p≪0.0001	—
	**Effect size**	0.28	0.26	0.38	−0.09	0.002	—

Table 1 shows the mean attenuation coefficient and standard deviations for both regions. Similarly to Figs. 2(e) and 2(f), the tissue attenuation coefficients in region 1 are lower than in region 2; for example, the “fresh” method has a tissue attenuation of $2.1\pm 1.1\;{\mathrm{mm}}^{-1}$ (mean ± standard deviation) in region 1, whereas the tissue attenuation in region 2 was $2.5\pm 1.0\;{\mathrm{mm}}^{-1}$.

Different trends are observed among the sample handling methods depending on the chosen region. In the first region, a resemblance between the “fresh” handling method and the “snap” is observed based on the effect size (δ=0.008). However, in the second region, both the “snap” (δ=−0.09) and “formalin” (δ=0.002) samples exhibit negligible effect sizes compared with the “fresh” sample. This suggests that the “snap” sample closely resembles the “fresh” sample in both regions, whereas the “formalin” sample only shows similarity in region 2. Nevertheless, despite the very small effect sizes, both preservation methods yield statistically significant differences relative to the “fresh” sample (p<0.0001).

### 3.2 Morphological Observations

**Fig. 3:**
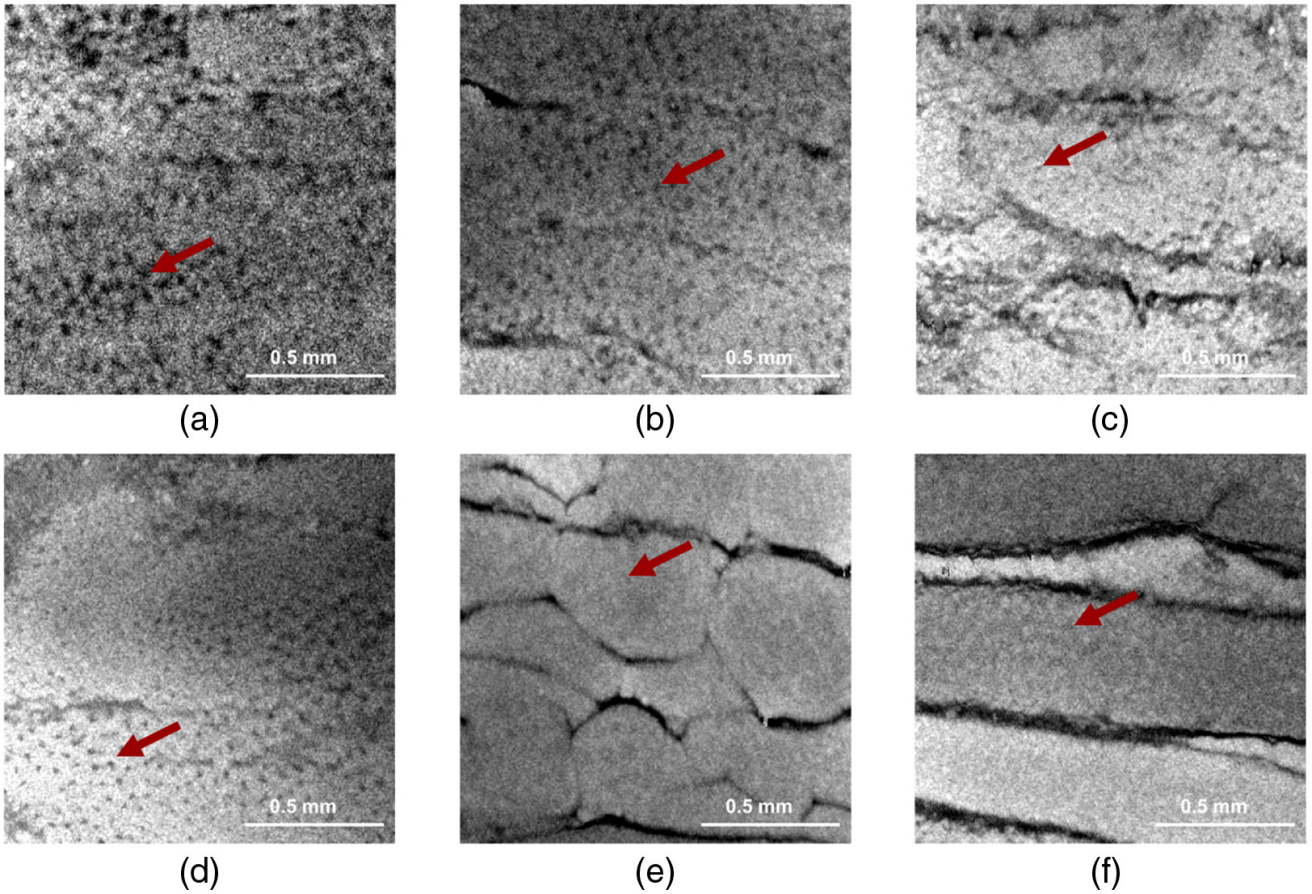
Incoherent averaged OCT *en face* view images taken in the depth range of 15 to 50  μm inside the colon (region 1). A crypt is indicated on each of the images using a red arrow. The scalebar is 0.5 mm. (a) Direct. (b) Slow. (c) DMSO. (d) Snap. (e) Formalin. (f) Fresh.

Figure 3 illustrates the incoherent averaged OCT *en face* view images at a depth range of 15 to 50  μm from the sample surface. Red arrows indicate crypts visualized as dark spots. By comparing the crypt size among the sample handling methods, it is revealed that frozen preparation methods seem to have larger crypts compared with the “formalin” and “fresh” methods. However, complete quantification is challenging due to the small crypt size for the “fresh” and “formalin” samples.

Macroscopic alterations can be observed as dark curved lines and are visible even after the surface is flattened. These lines are considered independent of the preparation methods as they are longitudinal mucosal folds.^26^^,^^29^^,^^30^ The longitudinal mucosal folds vary from sample to sample in both size and number of folds within the extracted area. It is notable, however, that the “formalin” sample seems to have more of these folds compared with the rest of the samples.

**Fig. 4:**
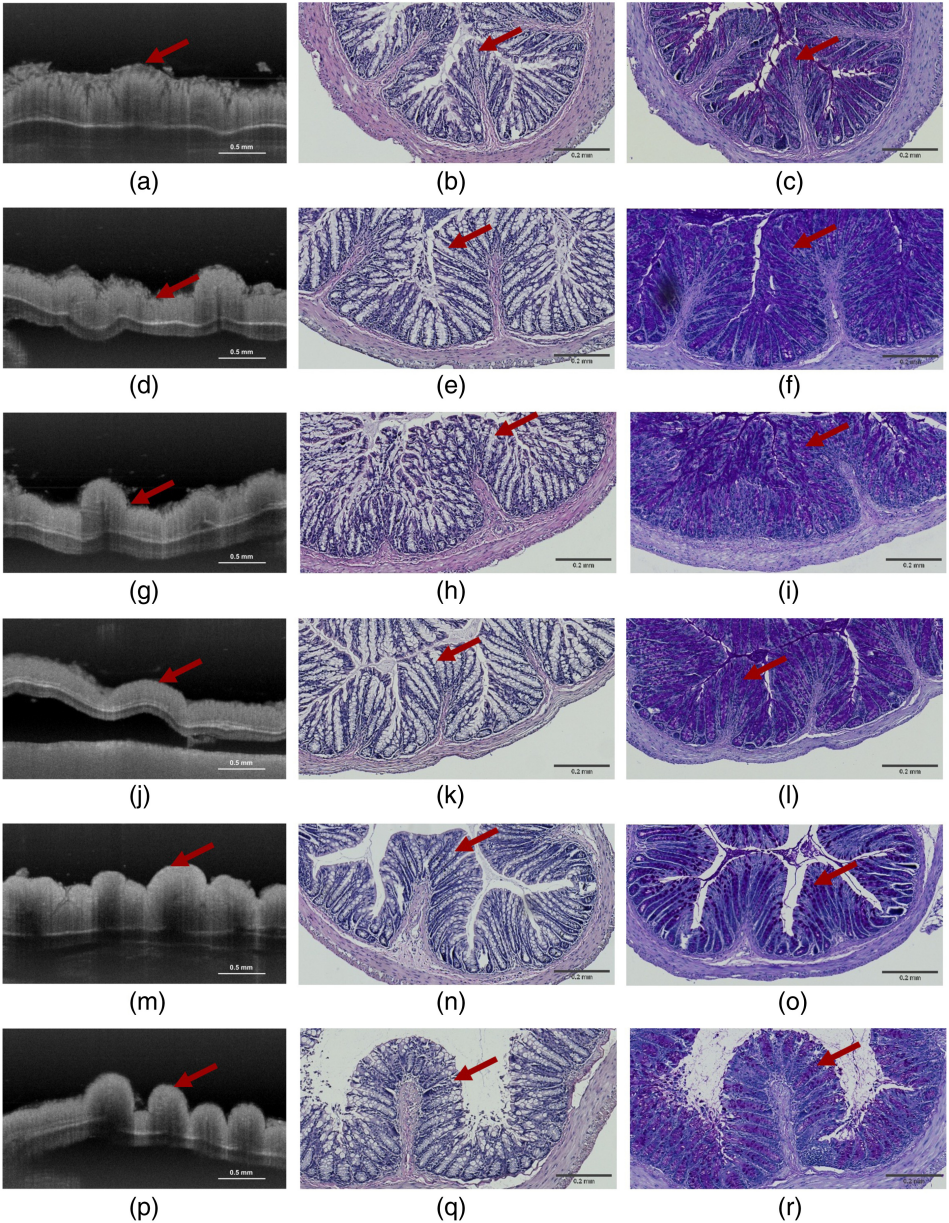
OCT B-scans and histology. Left side: 11 incoherently averaged OCT B-scans over 38  μm. Middle: H&E-stained tissue. Right side: PAS-stained tissue. Red arrows indicate the crypts surrounded by goblet cells. The scalebar is 0.5 mm for the OCT images and 0.2 mm for the histology images. (a)–(c) Direct. (d)–(f) Slow. (g)–(i) DMSO. (j)–(l) Snap. (m)–(o) Formalin. (p)–(r) Fresh.

Images of morphological structures, including an OCT B-scan and representative histology, can be seen in Fig. 4. The histological images of the “fresh” sample show slight breakage at the beginning of the epithelium, exhibiting tissue degradation, which may have been incurred due to being submerged in PBS for 2 h to emulate the time for transporting and imaging the “fresh” sample with OCT. The “formalin” sample shows the least degradation as it was fixed immediately after extraction. The goblet cells are clearly observed as dark purple circles placed along the crypts in the PAS-stained slide in the “formalin” sample [Fig. 4(r)]. These goblet cells are an important indication of the structural condition of the tissue as they change in the other sample handling methods.

In the “direct” sample, the goblet cells are observed to be missing or broken apart. All images of this sample show a frayed epithelium layer and a clear enlargement of the crypts [Figs. 4(a)–4(c)]. This preparation method appears in the morphological images to be the most damaged compared with all the other sample handling methods. For example, although the “slow” sample does show a degradation of the epithelium layer, it is not as pronounced as for the “direct” sample. The “snap” sample only has small changes in the epithelium and displays the best morphological structures of the freezing methods.

## 4 Discussion

The results of our study reveal fundamental insights into the impact of sample handling protocols on the attenuation coefficients and morphology of colon tissue. The study quantifies changes in tissue attenuation associated with each sample handling method and relates these changes to structural changes in tissue using histology.

The two different regions tested (Fig. 1) indicate notable changes in the first region compared with the second region for almost all samples. In the first region, “fresh” most closely resembles “snap,” whereas in the second region, “fresh” bears a strongest similarity to both “snap” and “formalin.” A contrast was also observed in the percentage of nonphysical values; across all sample handling methods, 43% of the total attenuation coefficients in region 1 were negative and consequently rejected, compared with only 8% in region 2. By relating this difference in percentage of nonphysical values to the histological images (Fig. 4), there seems to be a correlation between the nonphysical values and areas of tissue with substantial fragmentation. Typically, these areas with substantial fragmentation are located around the crypts, likely due to, among other causes, the breakage of goblet cells. Goblet cells reside in the epithelium as unicellular intraepithelial mucin-secreting glands.^26^^,^^30^^–^^32^ Mucins are glycoproteins that naturally have a large water-binding capacity. Consequently, goblet cells will have a buildup of large amounts of water, which is likely to cause the observed fragmentation under the freezing conditions. When also considering the role of goblet cells in preserving and lubricating the epithelium’s surface, they are also more exposed to the freezing conditions.^26^^,^^30^^–^^32^ Freezing could cause crystallization in the intra- and extracellular matrix of the goblet cells, leading to their breakage and overall fragmentation. So, the percentage of nonphysical values could indicate goblet cell breakage and thereby function as a new metric for assessing tissue damage in future studies.

The formation of ice crystals in the intra- and extracellular matrix has been investigated for decades to assess the preservation of cells and tissue during freezing.^16^^,^^23^^,^^33^ Mazur et al.^33^ highlight the possibility of a link between the rate of freezing and the observed effects on cells in suspensions. In cell suspensions, cell lysis under low-speed freezing conditions appears to be partially due to highly concentrated cell solutions and cell dehydration, which leads to a mechanical interaction between the cells and the extracellular ice crystals. Low-speed freezing is primarily dominated by a change in concentration gradients, which induces dehydration of the cell.^17^^,^^18^ One approach to control the gradient and the environment inside the cell during freezing is to use a specially designed cryomedia. Cryomedia is composed of substances such as DMSO, which have a lower freezing point compared with water, thereby suppressing and controlling ice crystal formation within the cell.^17^^,^^18^ High-speed freezing, on the other hand, has an evident correlation between cell breakage and formation of intracellular ice crystals, which often happens in the plasma membrane and/or intracellular organelles.^33^

In our study, we tested both scenarios: high- and low-speed freezing on tissue. The “slow” method we investigated can be considered a low-speed freezing method, whereas the “direct” method is a high-speed freezing method. In both cases, we observed structural changes, especially in the epithelium layer. We hypothesize that changes in the epithelium layer could be due to mechanical stress induced by ice crystallization. Furthermore, the “direct” method displayed more tissue breakage compared with the “slow” method, which may indicate possible thermal shock damage induced by rapid temperature changes during the high-speed freezing process. We therefore tested whether cryomedia (cell culture media with 10% DMSO, selected based on common cell freezing protocols) would reduce the amount of tissue damage during the freezing process. The expectation was that it would prevent tissue breakage during freezing. Hence, the result of the tissue’s breakdown in the “DMSO” method was unexpected. Even more unexpected was the apparent increased tissue deterioration when compared with the “slow” sample without cryopreservation medium. There might be several reasons for this, such as time requirements for DMSO to diffuse into the tissue compared with a single cell and/or having used DMSO instead of other substances, e.g., glycerol, in the cryomedia. The differences in the effect of freezing in bulk tissue compared with cell suspensions have been investigated by Pegg.^15^ He concluded there was a correlation between the formation of ice crystals and tissue damage, and that current cryo-methods for cell suspensions needed further improvements in regard to controlling the formation of ice crystals in bulk tissues.^15^

The possibility of using snap freezing methods to preserve the structural integrity of the samples has also been explored in other studies. Li et al.^20^ found that snap frozen samples did not notably differ from fresh tissue samples in terms of attenuation coefficient; however, it should be noted that this study was conducted on nasopharyngeal tissue, which has a more homogeneous surface compared with colon tissue. The statistical difference (p<0.001) we observed might be due to the complex microstructure and large heterogeneity of cell types of colon tissue, which might have an increased likelihood of freezing damage.^15^^,^^21^ Further studies, including several tissues with varying structural properties, are needed to confirm this hypothesis. Still, we did obtain a negligible effect size (δ=−0.09), which indicates that “snap” might practically be similar to “fresh” and co-aligns with Li et al.^20^ and Kropatsch et al.^21^

Another parameter to consider is the transportation of the sample from the extraction site to the imaging site. This term encompasses both the duration of the transportation and the conditions under which the sample is stored during transit. In our work, the samples were stored in Eppendorf tubes and transported in an insulated container with ice. Using PBS, the degradation of the tissue was minimized, as seen in the histology images in Figs. 4(p)–4(r)]. This correlates with results presented in Hsiung et al.,^34^ where they showed that storing tissue in PBS postpones the tissue degradation for 2 h. The observed tissue degradation on the “fresh” sample in the outer layers might explain the observed differences between the “formalin” and “fresh” samples in region 1.

The “formalin” sample had the least amount of degradation and contained more longitudinal folds in the histological images compared with other methods. This was expected as the “formalin” sample was fixed right after dissection. Hsiung et al.^34^ also showed no tissue degradation on formalin-fixed hamster cheek pouch samples. In contrast to what we observed, they did observe an increase in muscle scattering and shrinkage of the epithelium, muscle, and connective tissue. The observation of shrinkage due to formalin fixation is a known phenomenon that may give rise to an increase in scattering. We did not observe an increase in scattering in our measurements, which could be due to differences in our formalin fixation procedure, e.g., using PBS rather than ethanol. Ethanol is normally used for dehydration of the tissue, which can create shrinkage.^35^^,^^36^ Still, to address the effect of the formalin fixation on the tissue, it would be interesting, in a future study, to combine the measurements of layer thickness with the extracted attenuation coefficient to further describe how samples are affected by the sample handling methods.

It is important to highlight that this study focused on identifying the optical properties of the tissue and not on biochemical changes, which have been shown to be less affected by the freezing procedures.^37^^,^^38^ It may be that other sample handling methods should be considered if both structural and biochemical properties must be preserved.

In our study, we only considered the overall attenuation coefficient of the tissue, which comprises both scattering and absorption. In OCT-based studies of tissue attenuation, it is common to approximate that the attenuation coefficient is equivalent to the scattering coefficient as the absorption coefficient is typically several orders of magnitude smaller.^6^^,^^39^ Some models exist that can independently fit the scattering and absorption coefficients from OCT data^9^ and could be considered for future investigations. However, it is expected that the same trends will be observed as those we present here.

Other spectroscopic imaging modalities, such as laser spectroscopy, can also be used to independently extract scattering and absorption coefficients, based on tissue diffusion, transmission, and reflectance.^40^^–^^42^ It might be interesting to explore whether our results hold for other imaging modalities, and in particular, whether there is a difference in the optimal sample handling method to preserve either scattering or absorption properties.

## 5 Conclusion

Our analysis showed that none of the handling methods can replace the use of fresh colon tissue when measuring the tissue attenuation coefficient. However, in the case of fresh tissue being unavailable, formalin-fixed and snap frozen tissue samples yield the best alternative with negligible effect sizes. This result is supported by the measured attenuation coefficients (formalin: $2.5\pm 1.3\;{\mathrm{mm}}^{-1}$, snap: $2.8\pm 1.4\;{\mathrm{mm}}^{-1}$ versus fresh: $2.5\pm 1.0\;{\mathrm{mm}}^{-1}$) and the morphological observations with histology tissue staining.

This systematic study highlights and emphasizes the importance of understanding and characterizing the sample handling method for a given experiment as each of the six methods we considered had different effects on the colon tissue. All freezing methods showed a change in attenuation coefficient and degradation of goblet cells, albeit to different degrees. The results also demonstrate the challenges in comparing the outcomes from different studies when different sample handling methods are applied and underscore that sample handling is a crucial consideration when developing methods for translation to *in vivo* measurements, especially for diagnostic purposes.

## Data Availability

Data are available from the authors upon request.
